# Relationship between persuasive metadiscoursal devices in research article abstracts and their attention on social media

**DOI:** 10.1371/journal.pone.0231305

**Published:** 2020-04-13

**Authors:** Cindy Sing-Bik Ngai, Rita Gill Singh

**Affiliations:** 1 Department of Chinese and Bilingual Studies, The Hong Kong Polytechnic University, Hong Kong SAR, China; 2 Language Centre, Hong Kong Baptist University, Hong Kong SAR, China; University of Albany, State University of New York, UNITED STATES

## Abstract

Research article abstracts often convince readers that the article is worth reading. Therefore, they rely not only on the quality of arguments or novelty of findings to persuade readers but also linguistic markers in the form of metadiscourse to assert a position on an issue, increase readability of a text, engage readers, and avoid objection to the writer’s interpretations, thereby enhancing the credibility of the text. Given that research article abstracts are often published online and their newsworthiness would affect whether they would be ultimately read, Altmetric.com, which emerged in 2010, can help quantify the popularity of research article abstracts by counting views on social media and other platforms such as news and policy documents. Yet a study on how metadiscoursal devices are used to persuade readers, and how they are correlated with Altmetric Attention Score (AAS) provided by Altmetric.com, merits attention. In our study, we examined 241 abstracts from 50 top journals in 12 disciplines with the highest AAS from 2014–2018 and performed a quantitative analysis of the interactive and interactional metadiscourse markers exhibited in the abstracts. Overall, we found a positive correlation between the use of metadiscourse and AAS. Furthermore, we noticed that each discipline used distinct metadiscourse markers in abstracts with high AAS, which contributed to its respective discipline-specific conventions. It has been previously shown that the use of an array of interactive and interactional metadiscourse renders the abstract more worthy of attention. Being knowledgeable of rhetorical choices in relation to metadiscoursal devices will enable writers to construct more persuasive abstracts by making informed judgments about the appropriate use of metadiscourse to draw the attention of readers in their respective disciplines.

## Introduction

Scientists nowadays tend to promote their work to the audience [1] and persuasive features in academic communication are vital [2, 3]. Researchers have also been increasingly expected to engage not only experts of their own disciplines, but other disciplines, and sometimes even with the scope of interest being extended to non-experts, such as policy-makers and the general public [4, 5]. Writing persuasively, particularly the abstract, has become crucial to engage the audience since researchers have to disseminate findings to a broader audience through the use of social media and widespread adoption of electronic publishing [6]. Apart from the obvious role of summarizing the research, the abstract of a research article also plays a crucial role in grabbing the attention of readers and appeals to them that the article is worth reading [4, 7, 8].

Due to the vital role of the abstract which is meant to persuade readers to continue to read the article, the way as to how persuasion is achieved is worth examining. According to Petty and Cacioppo’s [9] Elaboration Likelihood Model, persuasion that is structured on the quality of arguments or content is referred to as persuasion through the central route while persuasion that is based on “peripheral cues” such as language use is coined as persuasion through the peripheral route. Pierro, Mannetti, Erb, Spiegel and Kruglanski[10] and Blankenship and Holtgraves [11] found that both content/quality of arguments and peripheral cues (i.e. linguistic markers) may exert a persuasive effect on the audience. In a similar vein, we contend that while content in the text is important [12], linguistic markers in abstracts might be able to determine whether the audience will read the article as academics do not only create texts that represent an objective external reality, but employ language to “construct and negotiate social relations”[13, p.66]. In particular, linguistic metadiscourse markers facilitate readers to understand and interpret the text so as to provide a credible interpretation of the research, consider alternative interpretations and acknowledge the writer’s awareness of his/her readers [13, 14]. In other words, metadiscourse is an essential persuasive aspect of academic discourse and an important means of facilitating communication, increasing readability of a text, building rapport with and engaging readers, and avoiding rejection of the writer’s interpretations[13, 15, 16]. It is a characteristic of a variety of languages and genres[14, 17].

Considerable research has been conducted on the use of metadiscourse markers by different language, cultural or disciplinary communities. Many studies [18–22] conducted a comparison of the use of Hedges and Boosters by writers from various cultures and drew a conclusion that cross-cultural/linguistic differences are evident in the use of such markers. In addition, the use of metadiscourse markers such as Boosters for persuasion has been investigated in multiple science disciplines[4], while the use of a variety of markers in the abstracts of Turkish and American students’ MA theses has been examined with noticeable variations observed[23].

However, a study on the persuasive effect of the use of metadiscourse, particularly in terms of how a variety of metadiscourse markers to persuade readers is correlated with AAS, is non-existent. This article seeks to contribute to a profound understanding of the relationship between metadiscourse markers and AAS, which is indicative of the popularity or persuasiveness of research articles on new and social media platforms and can be accessed by a large audience, potentially having a far-reaching societal impact [24]. Since the persuasive function is realized in metadiscourse while AAS reveals the popularity of research articles, we posit that there will be relationship between them. The variation in the use of metadiscourse markers in research article abstracts with high AAS will provide useful knowledge of how to create abstracts to gain the attention of the intended audience. Some light can also be shed on the variations in the use of such markers in different academic disciplines, thereby helping writers make decisions about the appropriate use of metadiscourse in their specific disciplines.

## Conceptual framework

### Metadiscourse

Studies have confirmed that metadiscourse is a vital feature of persuasive and argumentative discourse and people make choices on using linguistic devices to interact with others in different genres and disciplines[13, 25, 26]. For example, Crismore and Farnsworth [27] highlighted that the use of Attitude Markers and Hedges and Boosters could increase the persuasiveness of science texts. Abdi [28] concluded that Boosters and Hedges could be used to establish credibility in research articles in the social sciences and science fields, while Afros and Schryer [29] found that Self-mentions could attain this aim. Virtnanen [30] confirmed that editors of newspapers employed Hedges and Boosters when convincing readers of survey findings, and Hyland [31] noted that CEO letters used Evidentials, Hedges and Boosters to persuade their stakeholders.

A wide range of metadiscourse taxonomies has been put forward[16, 27, 32, 33]. We drew on Hyland’s [13, p.26] taxonomy of textual and interpersonal functions of metadiscourse using interactive and interactional types respectively which clearly identify rhetorical functions and reduce overlapping functions. Interactive metadiscourse is defined as devices which help to organize a text coherently by linking sentences to each other and other texts so that readers can understand it better. The way they are used relies on the writer’s knowledge of what to include for the academic community and his/her understanding of the readers. There are five kinds of interactive metadiscourse[13]. Transitions (e.g. *but*, *and*) facilitate readers to interpret connections between ideas by indicating how the writer thinks. Frame Markers explicitly refer to elements of text structure or sequences, indicating changes in the flow of the context. For example, sequencing phrases (e.g. *First*, *then*), concluding phrases (e.g. *to conclude*, *in summary*), discourse goals (e.g. *My aim is…*, *we try*) and a shift of topic (e.g. *well*, *now*) come under this category. Endophoric Markers are phrases that refer to information in other parts of the text (e.g. *as seen above*). Evidentials refer to information from other texts. In this sense, citations (e.g. *According to…/A states that…*) facilitate the reader’s interpretation of the text and indicate previous work on the subject, thus strengthening the persuasiveness of the text. Code Glosses are defined as the use of phrases to elaborate or explain information, and words like *such as*, *in other words*, *for example* are used.

Interactional metadiscourse is evaluative and shows the writer’s stance and attitude[13]. There are five types of interactional metadiscourse[13]. Hedges indicate tentativeness of the findings to reduce criticism from peers in the community[13, 34, 35], and include words like *may*, *probably*, *possible*. Boosters emphasize certainty of claims to obtain peers’ acceptance of claims and consist of phrases such as *prove*, *definitely*, *of course*. Attitude Markers indicate the writer’s attitude towards the text with the use of words such as *surprisingly*, *I agree*. Engagement markers build relationships with the reader by addressing the reader (e.g. *‘you can see’*). The use of second person pronouns, imperatives and question forms are evident in relational markers. Self-mentions refer explicitly to the author in the text and are reflected in the frequency of the use of first person pronouns (e.g. *I*, *we*, *my*). This categorization system is detailed and grounded on data [13]. It also helps to identify the functions that writers use and compares the persuasive patterns used by academic communities in different disciplines.

Metadiscourse is intricately tied to the norms and expectations of the professional community [26, 31]. Writing research article abstracts involves using metadiscourse that will persuade readers of that particular academic community. Therefore, different academic disciplines use different metadiscoursal devices to achieve their communicative intentions [4, 36, 37] and writers from different disciplines are required to follow certain conventions when presenting their claims[38]. Differences between the use of interactional metadiscourse in soft and hard disciplines [26, 31, 39] and interactive metadiscourse have been found[37, 40–42]. For example, Hyland [37] found that the science and non-science disciplines varied in their use of Code Glosses in that the hard disciplines used Code Glosses for reformulation but the soft disciplines employed them for elaboration. Peacock [42] conducted a study on the use of linking adverbials (e.g. *nowadays*, *thus*), which makes the relationship between two units of discourse clear, similar to Hyland’s transitions, in 320 research articles in eight disciplines and concluded that science disciplines used fewer linking adverbials when compared with the non-science ones.

### Metadiscourse and research article abstracts

Research articles are the main means by which researchers disseminate their latest scholarly work and demonstrate the novelty of their scholarly undertakings. With researchers being required to publish more alongside the subsequent monumental rise in the number of publications available on new and social media platforms, the abstract of a research article is integral to readers in facilitating them to choose useful articles to read that are related to their expertise [43–45]. The abstract has to draw readers’ attention through explaining the significance of the study, the originality and as well as demonstrate professional credibility and academic community membership of a particular discipline[7, 46]. In writing abstracts, academics do not only create texts that represent an objective external reality, but employ language to “construct and negotiate social relations”[13, p.66]. This requires the use of metadiscourse to enhance persuasiveness in order to engage readers, provide a credible interpretation of the work, acknowledge alternative interpretations or objections and the writer’s awareness of his/her readers [13, 15, 16].

Previous studies have investigated the features of abstracts such as the use of rationalization strategies as realized in the move structure, and the use of Boosters for persuasion as well as Formalization linguistic features [4]. The employment of different metadiscoursal devices in the abstracts of Turkish and American students’ MA theses has also been examined [23]. Due to the importance of research article abstracts in captivating readers’ attention, we postulate that an effective use of metadiscourse in abstracts could enhance the persuasiveness of the article and draw more public attention, especially on the new media platform. Furthermore, we argue that each distinct academic discipline would use different metadiscoursal devices to strengthen the persuasiveness of their research article abstracts. As a result, we investigated the effect of the use of different metadiscoursal devices in Altmetric Top ranking articles from diverse academic disciplines, and the correlation between their use and AAS because these devices are primarily used to negotiate meanings in texts and engage readers[13].

### Altmetrics and the importance of gaining public attention

Citation indexes have been used as a method of measuring the impact of research publications in different fields [47–49] and the higher the number of citations, the more scholarly impact an article is assumed to have. However, weaknesses of citation indexes include that they do not take into account the reasons the article is cited (e.g. as criticism), there may be long delays from publication to placement in citation databases[50], and most importantly, they do not show the impact of the article beyond the academic research community [51, 52].

Due to the accelerated use of social media and electronic publishing [6, 53, 54] which allows one to promptly disseminate the most recent research findings, even before publication[24], Altmetrics (a combination of the words *alternative* and *metrics*), one of the bibliometrics, was created to measure the impact of published research promptly beyond traditional citations. The advent of social media has further expanded the access of articles to a broader audience so as to gain more public attention. Altmetrics quantify the popularity of a research article by calculating counts on a number of platforms such as websites, blogs, and posts on social media platforms including Twitter, Sina Weibo, LinkedIn, and Facebook and other platforms like Mendeley, news outlets, and Wikipedia [6, 47, 53, 55, 56]. All the online tweets, shares, likes, and tags are converted into an aggregate score[57].

Altmetric Attention Score (AAS) aka Altmetric score provided by Altmetric.com is one of the most prominent altmetric data providers in measuring public attention and interest of academic articles on new media and social media [58]. Altmetric.com contains data on over 2.6 million papers published from 2011 onwards[59]. AAS is currently used by many publishers and organizations such as Genetics Society of America, PLoS, Springer Nature, Taylor & Francis Group, Wiley, and University of Michigan Press to reflect the quantity (higher attention, higher score) in conjunction with quality (weighting given based on different sources) of attention received by each research article. AAS is indicative of public attention and potentially shows the impact of research on society, while extending the concept of scientific impact to other kinds of impact (e.g. societal), which are often disregarded by traditional forms of impact assessment[50, 60].

However, altmetrics may lack credibility [60] and AAS has been criticized as a problematic indicator in the altmetric community since the wide range of social media sources included is of concern to the academic community [61], and it is possible that articles can receive a high AAS due to a variety of reasons. Newer articles might obtain a higher AAS than older ones[6]. Studies have therefore been done to examine the relationship between traditional citation-based metrics (i.e. citation counts) and altmetrics. Shuai et al.[62], Eysenbach [63] and Peoples et al. [64]noted a positive correlation between Twitter mentions, the number of tweets and number of citations. Priem et al. [65]found a moderate correlation between Mendeley and Web of Science citations. Similarly, Li et al. [66]found that the citation number from Google Scholar was positively associated with the number of Mendeley readers, and Thelwall et al. [24]found a linkage between higher metric scores and higher citations for PubMed articles with positive AAS in six out of the 11 metrics. Other studies found Mendeley readership counts have a strong association with citation counts [67–70] while Huang et al. [47] found that AAS is positively associated with higher citations for six PLoS journal articles. Yet an overreliance on data sources such as Twitter and Mendeley to determine the scores in AAS has raised scientists’ concerns [58].

Despite its limitations, altmetrics encapsulate a wider view of research visibility to evaluate the impact of a research article in society and the particular disciplinary community for a broader audience[71, 72]. Haustein et al. [73]and Araujo et al. [74]concluded that altmetrics should be viewed as complementary to citations instead of an alternative and altmetrics may reflect public/societal interest instead of interest from the scientific community. Altmetrics provided by Altmetric.com, which is well-recognized for its high quality and richness of data collected [58], is a useful measurement employed in disseminating research via social media [6, 47] and the attention from social media is likely to increase the chances for citations. Costas, Zahedi, and Wouters [75] pointed out that articles in the social sciences, humanities, medical and life sciences fields had higher altmetric scores, reflecting societal impacts such as health and government policies.

With the growing importance of almetrics, we wanted to examine whether AAS is correlated with persuasive writing in research article abstracts. As discussed in the previous section, metadiscourse is often employed to enhance the persuasiveness of research article abstracts, particularly because not all research articles are made available publicly. For those non-open access articles, readers from the non-academic community are only able to read the abstract on new and social media platforms. As a result, it is likely that a persuasive research article abstract may help draw more public attention on such platforms. To date, there is hardly any study on how AAS is linked with the use of metadiscoursal devices in research article abstracts to persuade readers. We posit that as the persuasive function is realized in metadiscourse while AAS indicates the popularity of research articles, there might be a relationship between the two.

### Purpose of study and research questions

Prior research on metadiscourse has focused on academic, newspaper, and business genres, primarily investigating the usage of different metadiscoursal devices in academic articles or other genres[26, 28, 31, 37, 39, 42, 76]. Since there is a dearth of research on the effects of the use of metadiscourse in realizing persuasion in abstracts and most importantly, its association with the AAS, which has gained prominence in recent years, we conducted this study to address and bridge this gap. AAS is an indicator of public attention while metadiscourse engages readers, so AAS is expected to be correlated with the use of metadiscoursal devices in abstracts. The audience of research article abstracts on social media comprises not only researchers from specific disciplines but also the public, non-experts and officials[5, 77], so the necessity of capturing attention through the use of metadiscoursal devices is of integral importance.

We scrutinized 241 research article abstracts from 12 academic disciplines with the highest AAS scores from 2014–2018 to reveal their use of metadiscoursal devices and correlation with the AAS. The focus was on identifying whether the frequency in the use of metadiscoursal devices is associated with AAS, which devices are associated with AAS in different academic disciplines, and the variation in the usage of such devices in research article abstracts with high AAS from 2014–2018. Given the contribution and weighting of news, blogs, Twitter, and Facebook on altmetric scores [58, 78], we also scrutinized the correlation between the use of metadiscoursal devices and news, blog, Twitter, and Facebook mentions in AAS.

Therefore, the following research questions were derived:

RQ1a: Was there a relationship between the use of metadiscourse and AAS?

RQ1b: Was there a relationship between the use of metadiscourse and the number of news, blog, Twitter, and Facebook mentions in AAS?

RQ2a: Was there a relationship between the use of metadiscourse and AAS in different disciplines?

RQ2b: Was there a relationship between the use of metadiscourse and the number of news, blog, Twitter, and Facebook mentions in AAS in different disciplines?

RQ3: What was the pattern of the use of metadiscourse with high AAS in research article abstracts from 2014–2018?

## Materials and methods

### Sampling

To perform a longitudinal analysis of the use of metadiscoursal devices in the top AAS articles, we collected research article abstracts of Altmetric Top 50 published by Altmetric.com for five consecutive years from 2014 to 2018. There were two reasons underlying the sample and period of selection: 1) the Top 50 articles instead of Top 100 were chosen because the AAS for Top 100 articles produced a flattening curve after 50 which meant that a small difference of AAS was observed in the articles that ranked from 50 to 100; and 2) although the Top 100 articles were published since 2013, the AAS of the articles in the awarded year was only made available from 2014 onwards. As a result, we selected Altmetric Top 50 from 2014 to 2018 for a longitudinal analysis of the use of metadiscoursal devices. After filtering out those articles without an abstract, we included 241 research article abstracts (2231 sentences/60,456 words) from 12 disciplines, namely Biological Sciences, Chemical Sciences, Earth (and Environment) Sciences, Engineering, History & Archaeology, Information and Computing Sciences, Material Sciences, Medical and Health Sciences, Physical Sciences, Psychology and Cognitive Sciences, Research and Reproductivity, Studies in Human Society, in our data pool.

### Metadiscoursal analysis

For the examination of metadiscoursal devices in the selected research article abstracts, we adapted Hyland’s [13]interpersonal model, a widely recognized model for metadiscourse analysis[4, 19, 46, 79, 80]. This model consists of the taxonomy of both interactive and interactional metadiscourse devices that emphasize the interaction between the writer and reader in the production and understanding of the text. See Table 1 for the interactive and interactional types of metadiscourse adapted in our study.

**Table 1:** Interactive and interactional types of metadiscourse adapted.

Types (Coding code)	Description	Examples
**Interactive**		
Transitions (T)	Express relations between main clauses	in addition; but; thus
Frame markers (FM)	Refer to discourse acts, sequences, or stages	Firstly, finally; to conclude
Code glosses (CG)	Elaborate propositional meanings	that is; in other words
Endophoric markers (EdM)	Refer to information in other parts of the text	noted above; see Table X
Evidentials (E)	Refer to information from other texts	according to X; Y argued
**Interactional**		
Hedges (H)	Withhold commitment and open dialogue	may; perhaps
Boosters (B)	Emphasize certainty or close dialogue	certainly; it is clear that
Attitude markers (AM)	Express writer’s attitude to a proposition	surprisingly; unfortunately
Engagement markers (EgM)	Explicitly refer to or build relationships with the reader	note that / you can see
Self-mentions (SM)	Refer explicitly to the author	I; we; my; our

Due to the multiple linguistic markers of the interactive and interactional types, we employed *Text Inspector*, an award winning text analysis algorithm developed by Prof. Stephen Bax[81], which has been used by major publishers including Cambridge University Press and students and staff at 145 universities and colleges in over 100 countries[82], for the automated analysis of the large data set collected. *Text Inspector* adapted Hyland’s taxonomy on metadiscourse analysis and is often used in research related to metadiscourse and EVP analysis[81, 83, 84]. However, as noted in a previous study, *Text Inspector*’s outputs are not “perfectly accurate”[81]. To ensure an accurate counting of metadiscoursal devices in the 241 research article abstracts collected, we conducted a four-stage text analysis in which both automated analysis [81] and manual checking [85] were employed to enhance the accuracy of marker identification and avoid the risk of overlooking other markers.

#### Stage 1: Automated analysis

We analyzed the 241 research article abstracts using *Text Inspector*. *Text Inspector* was able to detect over 300 metadiscourse markers from the ten types and four sub-types of interactive and interactional metadiscourse respectively as identified in Hyland’s study[13]. However, as found in a previous study, *Text Inspector* “counted all instances of discourse markers regardless of their appropriacies”[81:p.13]. As a result, we had to train a research assistant to review all *Text Inspector’s* output and the texts in the data pool.

#### Stage 2: Manual checking

A full-time research assistant with training in linguistics analysis was recruited and trained for two days to identify the metadiscourse markers of interactive and interactional types in 96 (40%) abstracts in 2017 and 2018. Then, the research assistant reviewed the *Text Inspector*’s results of the 241 abstracts within two weeks to check the markers identified by the software and locate the missing markers. The manual checking results were reviewed by the authors to confirm the changes needed. Through the human checking process, we found three main problems of the computer interpretation of the metadiscourse markers: 1) the software misinterpreted adjectives like first, second, third as Sequencing markers; 2) the software misinterpreted “well” as Topic Shift markers; and 3) the number of Boosters was rather limited. The first two problems were mainly related to the counting of Frame Markers in interactive metadiscourse, while the last issue emerged from the limited pool of Boosters included in the software.

#### Stage 3: Adjustments to the automated analysis

After manually checking the results, we adjusted the outputs of *Text Inspectors* in Frame Markers. We also included additional Boosters as mentioned in a previous study[4] to enlarge the number of markers in Boosters. All the outputs of *Text Inspectors* for all the abstracts were manually adjusted to improve the accuracy on the identification of metadiscourse markers (see Table 2 for the manually adjusted items in the *Text Inspectors* output).

**Table 2:** Manually adjusted items.

Types/ Year	2014	2015	2016	2017	2018
Frame markers: Sequencing	24	32	23	20	24
Frame markers: Topic shift	12	7	7	4	4
Boosters	19	18	16	18	19
Total	55	57	46	42	47

#### Stage 4: Data standardization

In order to examine the association of metadiscourse devices and AAS (RQ1 and 2) which had a very different corpus base, we standardized the raw count on the metadiscourse markers using z score for investigating the correlation between specific metadiscourse devices, AAS[86] and mentions from various sources including news, blogs, Twitter, and Facebook. Since the research article abstracts collected were of varied lengths, ranging from 25 to 1097 words, the comparison between markers count in the raw dataset (RQ3) could confound the results[81]. We therefore standardized the data using the “frequency per 100 words” [81]for ANOVA analysis. Subsequently, all statistical analyses were conducted on the standardized dataset.

### Statistical analyses

To answer RQ1 to 3, statistical analysis was conducted on the standardized dataset. For RQ1 and 2, Pearson product-moment correlation analysis was performed to 1) investigate the correlation between the use of metadiscourse and AAS, and 2) the correlation of metadiscoursal devices and AAS in research article abstracts from different disciplines. To ensure that the assumption of Pearson product-moment correlation was not violated, the test for linearity was also performed to check the linearity of the metadiscoursal devices and AAS. For the determination on the degree of the correlation, we adopted the criteria from Cohen’s study (1988) where r < .30 is defined as small effect size, r < .50 is a medium effect size and r>.50 is a large effect size[87: p.81]. With regard to RQ3, one way ANOVA was employed to reveal the different use of metadiscoursal devices in research article abstracts from 2014–2018.

## Results

### Association between the use of metadiscourse and AAS

Concerning RQ1a, we examined if there was a relationship between the use of metadiscoursal devices and AAS, so a Pearson product-moment correlation was run to determine the relationship between the use of metadiscourse and AAS (see Table 3 for the Pearson correlation results between metadiscoursal devices and AAS). There was a positive relationship with a small effect size found between the overall use of metadiscoursal devices and AAS, which was statistically significant (*r*(239) = .184, *p* = 0.004, *R*^2^ = .0338).

**Table 3:** Pearson correlation results between metadiscoursal devices and AAS.

				**Interactive**				**Interactional**							
		**AAS**	**T**	**FM**	**CG**	**EdM**	**E**	**Sub-total**	**H**	**B**	**AM**	**EgM**	**SM**	**Sub-total**	**Total**
	**AAS**	1	.167**	.191**	.178**	0.039	0.051	.190**	0.048	-0.049	-0.092	0.034	.145*	0.079	.184**
Interactive	**T**		1	.192**	.376**	-0.022	.211**	.982**	.242**	0.096	-0.015	.187**	.289**	.362**	.936*
	**FM**			1	.209**	0.009	-0.017	.270**	0.006	-0.033	0.074	0.042	0.081	0.062	.254**
	**CG**				1	-0.050	.137^*^	.455**	.207**	0.034	0.014	.284**	.255**	.352**	.492**
	**EdM**					1	-0.030	-0.011	-0.022	-0.022	-0.076	0.005	-0.007	-0.032	-0.020
	**E**						1	.355**	0.072	-0.002	0.089	0.036	.150*	.144*	.342**
	**ST**							1	.246**	0.085	0.006	.199**	.310**	.381**	.957**
Interactional	**H**								1	-0.022	0.076	0.111	.169**	.504**	.363**
	**B**									1	0.127	-0.060	.314**	.515**	.232**
	**AM**										1	0.028	0.111	.304**	0.099
	**EgM**											1	-0.075	.379**	.285**
	**SM**												1	.746**	.493**
	**ST**													1	.632**
	**Total**														1

* Correlation is significant at the 0.05 level (2-tailed)

** Correlation is significant at the 0.01 level (2-tailed)

We also found a positive correlation with a small effect size between the sub-total (ST) of interactive metadiscourse and AAS (*r*(239) = .190, *p* = 0.003, *R*^2^ = .036), which was statistically significant. Of the sub-types of interactive metadiscourse, we noted a statistically significant correlation between the use of Transitions (*r*(239) = .167, *p* = 0.009, *R*^2^ = .028), Frame Markers (*r*(239) = .191, *p* = 0.003, *R*^2^ = .0365), Code Glosses (*r*(239) = .178, *p* = 0.006, *R*^2^ = .0316) and AAS although the effect size was relatively small. As for the sub-types of interactional metadiscourse, we found a positive correlation between the use of Self-mentions and AAS, which was statistically significant (*r*(239) = .145, *p* = 0.025, *R*^2^ = .0209).

Regarding RQ1b, we found a positive correlation with a small effect size between the use of Transitions *(r*(239) = .193, *p* = 0.003, *R*^2^ = .0373) and the number of news mentions in AAS. Similarly, we uncovered a positive correlation with a small effect size between the use of Frame Markers (*r*(239) = .148, *p* = 0.021, *R*^2^ = .0219) and the number of Twitter mentions in AAS.

The small effect size but statistically significant correlation indicated a high amount of unexplainable variability in the data, which is very likely to have resulted from the different use of metadiscoursal devices by researchers from highly diverse disciplines. Therefore, further investigation into the relationship between the use of metadiscoursal devices and AAS in specific academic disciplines was warranted (RQ2).

### Variation in the use of metadiscourse in academic publications with high public attention from different disciplines

Due to the broad range of academic disciplines included in Altmetric Top 50 (see Table 4), we observed a high variability in the data resulting from the different use of metadiscoursal devices from different disciplines. To further explore the use of metadiscoursal devices from different disciplines and their relation with AAS and the number of mentions in news, blogs, Twitter, and Facebook (RQ2), we performed Pearson product-moment correlation on the use of metadiscoursal devices and its association with AAS in each discipline (12 in total). We found statistically significant correlations between specific metadiscoursal devices and AAS in three disciplines including Medical and Health Sciences, Studies in Human Society, and Information and Computing Sciences. Then we performed Pearson product-moment correlation to further reveal the use of metadiscoursal devices and the number of mentions in news, blogs, Twitter, and Facebook in these three disciplines.

**Table 4:** Distribution of research article abstracts in 12 disciplines from Altmetric Top 50 2014 to 2018.

Disciplines/ Years	2014	2015	2016	2017	2018	Total
1. Biological Sciences	11	9	6	13	1	40
2. Chemical Sciences	1	0	0	0	0	1
3. Earth (and Environment) Sciences	3	8	4	4	7	26
4. Engineering	1	0	0	0	0	1
5. History & Archaeology	0	1	1	4	4	10
6. Information and Computing Sciences	2	5	1	0	2	10
9. Material Sciences	0	0	1	0	0	1
8. Medical and Health Sciences	20	15	19	22	25	101
9. Physical Sciences	3	3	5	1	2	14
10. Psychology and Cognitive Sciences	6	0	0	0	0	6
11. Research and Reproductivity	0	5	2	1	1	9
12. Studies in Human Society	1	5	7	3	6	22

### Medical and health sciences

Although the abstracts from Medical and Health Sciences accounted for nearly half (42%) of our dataset, the Pearson product-moment correlation outputs were similar to the results for RQ1. There was evidence of a positive relationship with a small effect size between the total use of metadiscourse and AAS, being statistically significant (*r*(99) = .269, *p* = 0.009, *R*^2^ = .072).

**Fig 1:**
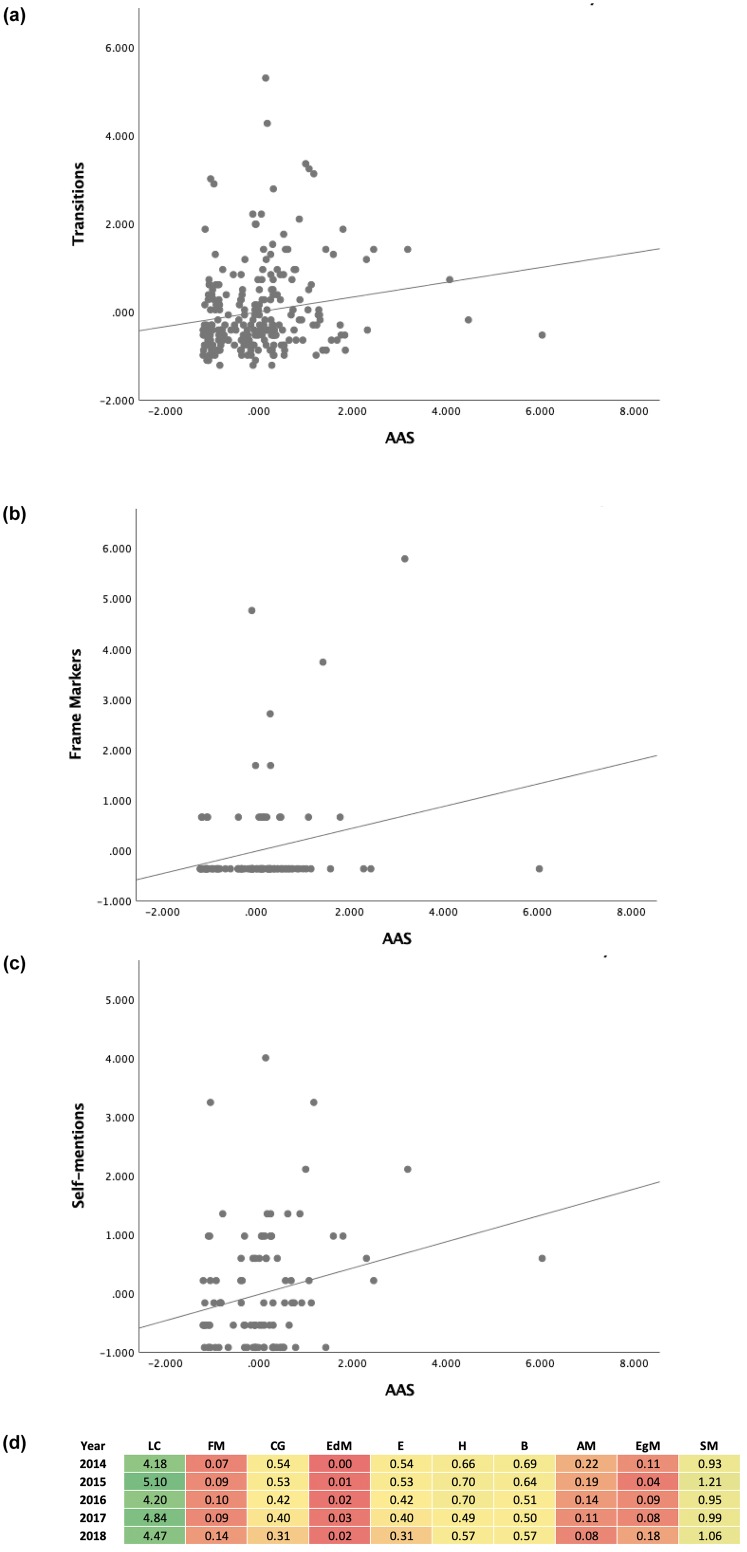
**a.** Scatter Plot with Fit Line of Transitions by AAS. **b.** Scatter Plot with Fit Line of Frame Markers by AAS. **c.** Scatter Plot with Fit Line of Self-mentions by AAS. **d.** Heat map illustrating the use of metadiscourse in Medical and Health Sciences abstracts (per 100 words) from 2014 to 2018.

A statistically significant positive correlation with a small effect size was observed in the sub-total use of interactive types of metadiscourse (*r*(99) = .263, *p* = 0.008, *R*^2^ = .069. In particular, evidence of a small effect size but positive relationship between the use of Transitions (*r*(99) = .245, *p* = 0.014, *R*^2^ = .06) and Frame Markers (*r*(99) = .229, *p* = 0.021, *R*^2^ = .0524) and AAS was noted. In addition, we found an association between Self-mentions and AAS in the interactional category, which was statistically significant (*r*(99) = .231, *p* = 0.02, *R*^2^ = .0533). See Fig 1a, 1b and 1c for the Scatter Plot with Fit Line of Transitions, Frame Markers and Self-mentions by AAS. Despite the fact that *r* was statistically significant for these three types of metadiscoursal devices, the small effect size implies that the strength of the relationship between the metadiscoursal features and AAS was rather weak which might have resulted from the wide variation in the use of metadiscourse in the Medical and Health Sciences discipline (see Fig 1d).

The examination of metadiscourse use and the number of news, blog, Twitter, and Facebook mentions in AAS revealed a statistically significant positive correlation with a small effect size observed between the use of Transitions and the number of news mentions in AAS (*r*(99) = .245, *p* = 0.013, *R*^2^ = .06). A positive correlation was also yielded between the use of Self-Mentions and the number of Twitter mentions in AAS (*r*(99) = .217, *p* = 0.03, *R*^2^ = .0471).

### Studies in human society

**Fig 2:**
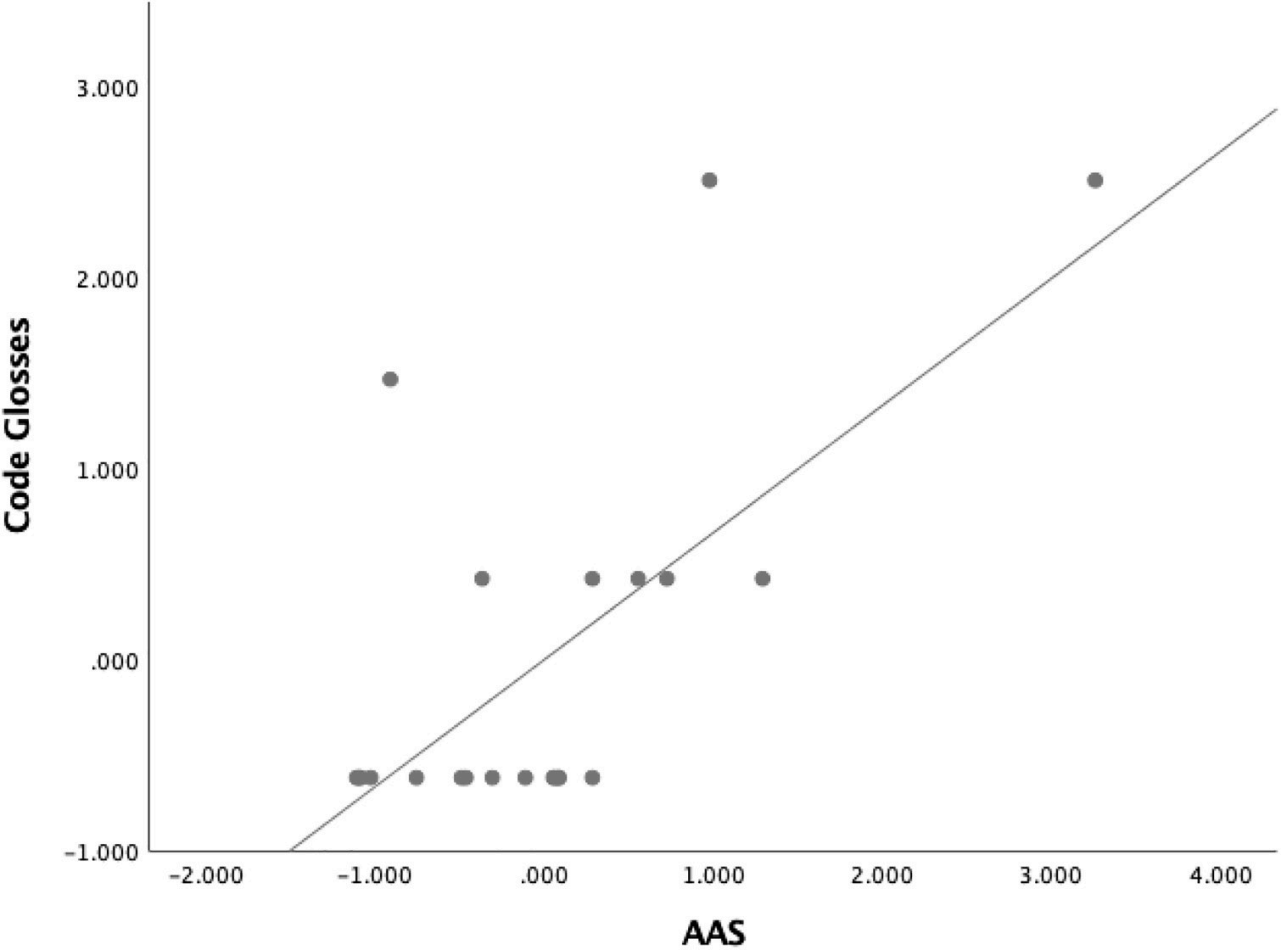
Scatter plot with fit line of code glosses by AAS in studies in human society.

We conducted Pearson product-moment correlation to determine the relationship between the use of metadiscoursal devices and AAS in Studies in Human Society, finding a positive correlation with a large effect size between the use of Code Glosses and AAS, which was statistically significant (*r*(20) = .666, *p* = 0.001, *R*^2^ = .443). See Fig 2 for the Scatter Plot with Fit Line of Code Glosses by AAS.

It is worth noting that a statistically significant positive correlation was witnessed between the use of Code Glosses and the number of news (*r*(20) = .560, *p* = 0.005, *R*^2^ = .314) and Facebook mentions in AAS (*r*(20) = .510, *p* = 0.013, *R*^2^ = .260). In addition, our analysis found a positive correlation between the use of Frame Markers and the number of blog mentions in AAS (*r*(20) = .438, *p* = 0.037, *R*^2^ = .192).

### Information and computing sciences

**Fig 3:**
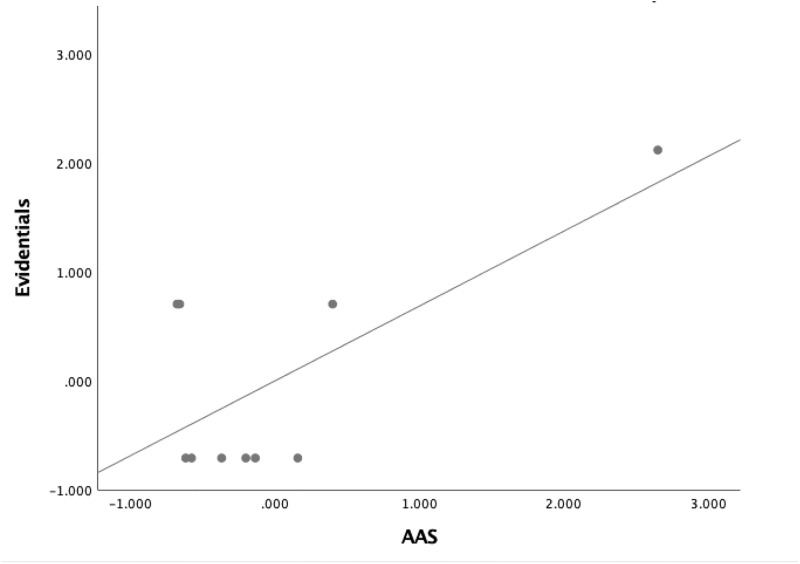
Scatter plot with fit line of evidentials by AAS in information and computing sciences.

Concerning abstracts from Information and Computing Sciences, the Pearson product-moment correlation result showed a positive relationship with a large effect size between the use of Evidentials and AAS, being statistically significant (*r*(8) = .687, *p* = 0.028, *R*^2^ = .473). See Fig 3 for the Scatter Plot with Fit Line of Evidentials by AAS.

The results from the investigation into the use of metadiscourse and the number of news and Twitter mentions were consistent with the above results. A strong positive correlation was found between the use of Evidentials and the number of news (*r*(8) = .662 *p* = 0.037, *R*^2^ = .438) and Twitter mentions (*r*(8) = .710, *p* = 0.022, *R*^2^ = .504) in AAS.

### The paradigm on the use of metadiscourse in papers with high public attention from 2014 to 2018

**Fig 4:**
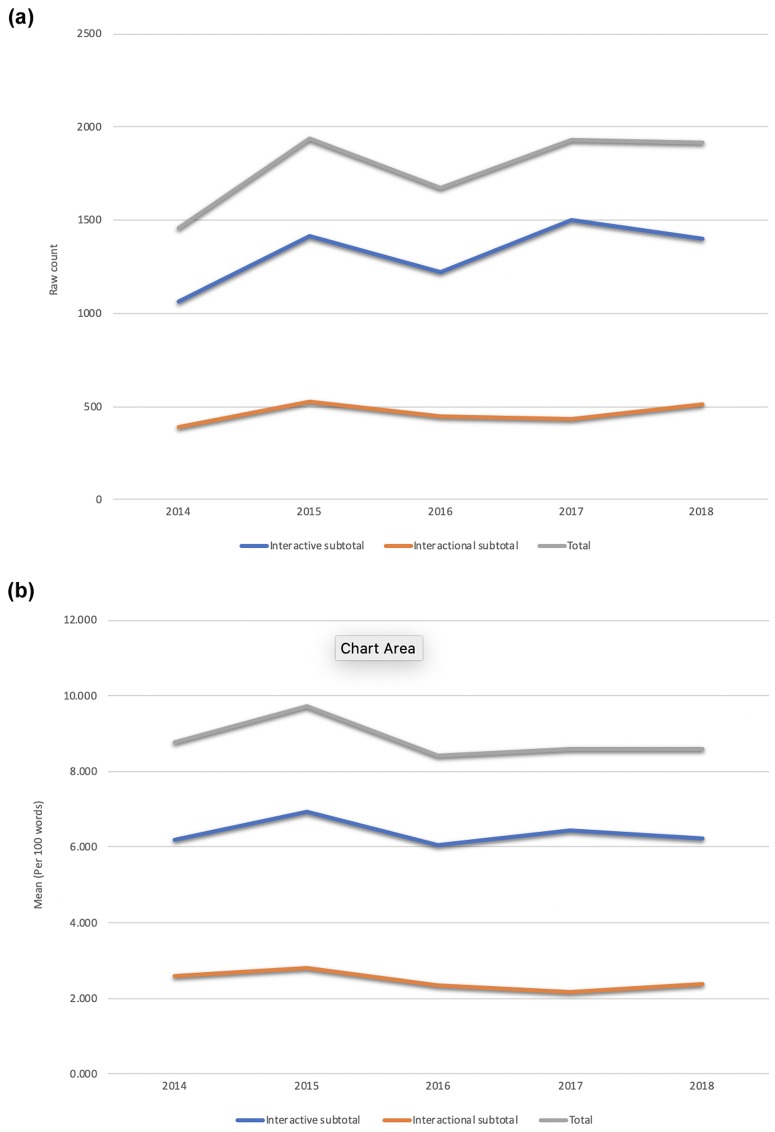
**a.** Total and subtotal count of interactive and interactional types of metadiscourse from 2014 to 2018 (raw count). **b.** Total and subtotal number of interactive and interactional types of metadiscourse from 2014 to 2018 (per 100 words).

In response to RQ3, although the raw count of metadiscourse in Fig 4a indicated a steady growth in the use of metadiscoursal devices in the past five years, the standardized data in Fig 4b reflected a relatively similar use of both interactive and interactional types of metadiscourse in the longitudinal study. However, the t-test results showed a statistically significant difference with a large effect size between the use of interactive and interactional metadiscourse over the period studied (*p*<0.0001, η_p_^2^ = 0.982), although the ANOVA results indicated that none of the specific metadiscoursal devices had statistically significant differences in their frequency of use from 2014 to 2018. In other words, the use of interactive types of metadiscourse was significantly higher than interactional metadiscourse over the five-year period.

**Fig 5:**
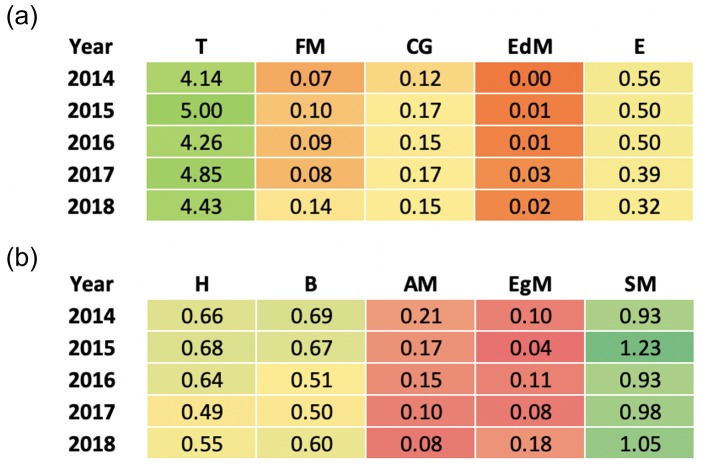
**a.** Heat map illustrating the frequency of interactive metadiscourse sub-types employed in Altmetric Top 50 from 2014 to 2018 (per 100 words). **b.** Heat map illustrating the frequency of interactional metadiscourse sub-types employed in Altmetric Top 50 from 2014 to 2018 (per 100 words).

Then we examined the use of different types of interactive and interactional metadiscoursal devices between 2014 and 2018. Transitions, Evidentials, Hedges, Boosters, Attitude Markers, and Self-mentions were frequently employed in the sub-types of interactive and interactional metadiscourse. A rising trend in the use of Transitions, Frame Markers, Code Glosses, Engagement Markers and Self Mentions was noted, whereas a clear falling trend in the use of Evidentials and Attitude Markers was found. See Fig 5a and 5b for the frequency of interactive and interactional metadiscourse sub-types employed in Altmetric Top 50 from 2014–2018.

**Fig 6:**
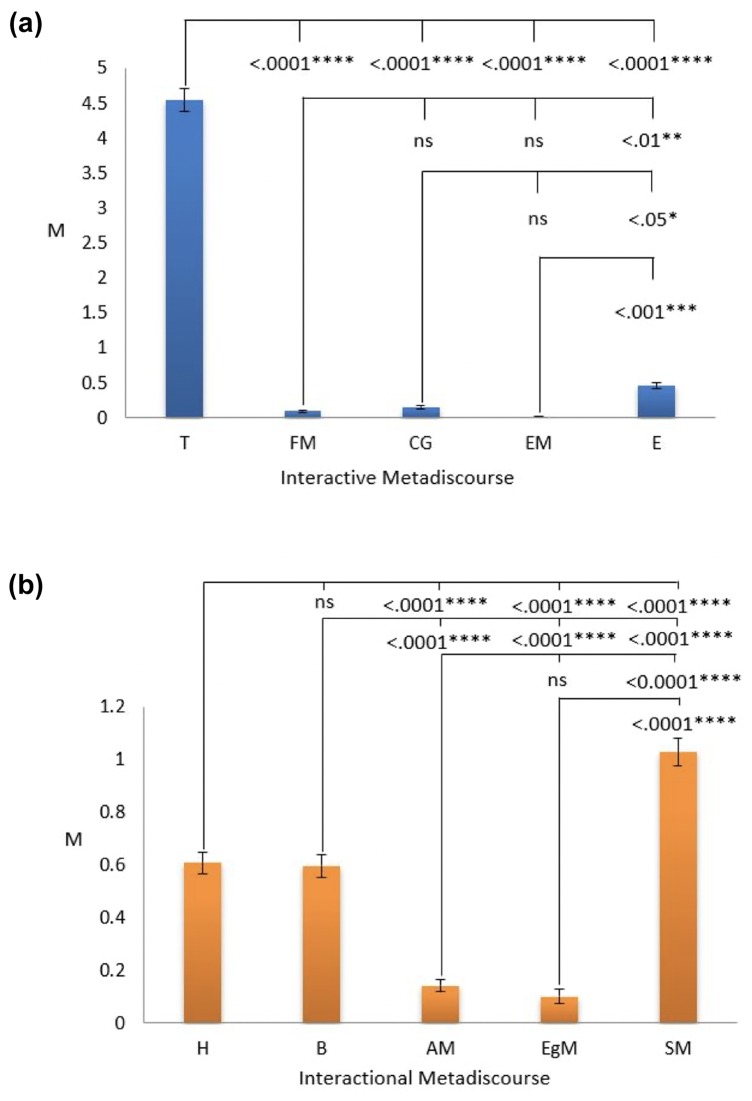
**a.** Different use of interactive metadiscourse sub-types in top AAS article abstracts. **b.** Different use of interpersonal metadiscourse sub-types in top AAS article abstracts.

ANOVA and Post Hoc test results indicated a statistically significant difference in the use of interactive metadiscourse sub-types (*F*(4,1200) = 645.766, *p*<0.0001). As shown in Fig 6a, Transitions and Evidentials were the most commonly used devices for interactive type of metadiscourse. Significant differences (*p*<0.0001) were witnessed between the use of Transitions (M = 4.539), Frame Markers (M = 0.095), Code Glosses (M = 0.152), Endophoric Markers (M = 0.014) and Evidentials (M = 0.454). Significant differences were also found between the use of Evidentials and Frame Markers (*p* = 0.009), as well as Endophoric Markers. (*p* = 0.001), CG (*p* = 0.045).

Apart from this, a statistically significant difference in the use of interpersonal metadiscoursal devices was noted (*F*(4,1200) = 94.864, *p*<0.0001). As depicted in Fig 6b, Self-Mentions, Hedges and Boosters were frequently used for interpersonal metadiscourse. Significant differences (p<0.0001) were found between the use of Self-mentions (M = 1.027), Hedges (M = 0.607), Boosters (M = 0.597), Attitude Markers (M = 0.143) as well as Engagement Markers (M = 0.101). Furthermore, significant differences (p<0.0001) were observed between the use of Hedges, Attitude Markers and Engagement Markers with Boosters being significantly used in comparison with Attitude Markers and Engagement Markers.

## Discussion

### Positive association between specific metadiscourse markers and AAS

The general results reveal that specific metadiscourse markers play a moderate role in the construction of persuasion in research article abstracts and are positively associated with AAS. To date, no studies have investigated the relationship between metadiscourse and AAS in Altmetric Top ranking research articles, and therefore, our study makes a contribution to the literature. Metadiscourse markers make the content of a text coherent, understandable and persuasive to readers, while encouraging readers to accept one’s claims[28, 39, 79]. In other words, metadiscourse markers serve a function of persuading and engaging readers[13], and similarly, AAS is indicative of research articles receiving high public attention and interest, and being persuasive [50, 60], and therefore, a statistically significant positive correlation though with a small effect size was found between the two.

A positive relationship between interactive metadiscourse markers: Transitions, Frame Markers, Code Glosses and AAS was revealed since these markers are employed to organize information in ways that readers would find it coherent, cohesive and convincing, with writers having to take into account readers’ knowledge, understanding, expectations and need for interpretive guidance [13, 28, 29]. Furthermore, our findings also revealed a significant correlation between Transitions and the number of news mentions in AAS as well as between Frame Markers and the number of Twitter mentions in AAS. Transitions are mainly conjunctions that facilitate readers’ understanding of the internal connections in arguments by adding, comparing, and sequencing, which are relevant to a text’s overall persuasive effect [13, 76]. Frame Markers signal the text structure, hence allowing readers to gain a better understanding of the flow of the text while Code Glosses provide additional information through rephrasing or elaboration to ensure that readers understand the significance of particular information in a way that the writer intends, reflecting the writer’s assessment of shared existing knowledge and understanding with readers, thereby fulfilling a persuasive function [13, 76].

With regard to interactional metadiscourse, an association between Self-mentions and AAS was found in Altmetric Top ranking articles. Self-mentions are the main way in which writers can promote a credible scholarly identity and garner support for their research claims through directly referring to themselves[13]. They play a vital role in mediating the relationship between the writer’s claims and their disciplinary community, enabling the writer to create an identity as both a member of the disciplinary community and an originator of novel ideas or important research value and impact [88].

Despite the statistically significant *p*-values found for RQ1 and RQ2, the Pearson correlation between specific metadiscourse markers and AAS had rather small effect sizes, indicating a low strength of relationship[89]. The effect size was small because the data set was diverse from 12 different disciplines in total, and as a result, the unique variations in each discipline might have lowered the correlation and effect size (see Fig 5a and 5b). There is also a possibility that metadiscourse markers were not frequently used in the abstracts of some disciplines which could have led to the lower correlations. A similar pattern was observed for the correlation between specific metadiscourse markers and AAS in the Medical and Health Sciences discipline which constituted 42% of the abstracts in our study. As has been found in a previous study [4], a significant number of differences in the structure, metadiscoursal and microdiscoursal features were evident in the research article abstracts from different science disciplines. As such, the high interdisciplinary variation in the use of metadiscourse could have affected the strength of the relationship between specific metadiscourse markers and AAS. Similarly, a high intradisciplinary variation in the use of metadiscourse might be expected in a broadly-defined discipline such as Medical and Health Sciences in which publications from a wide range of sub-disciplines including Biology, Psychiatry, Medicine, Health Science and Policy, Cardiology, Neurology, Oncology, Diabetes and Endocrinology are seen (see Fig 1d). Thus, the high variability of the sub-disciplines in the Medical and Health Sciences and different use of metadiscourse might have resulted in the small effect size even though the results were statistically significant.

### Disciplinary variations in the use of metadiscourse in publications with high AAS

One is likely to persuade readers of one’s scholarly claims if one frames the text using metadiscourse in ways that appeal to appropriate relationships in different disciplinary communities, which share disciplinary background knowledge, expectations and norms [2, 29, 39, 79]. In this respect, we found disciplinary variations in the use of metadiscourse markers in Altmetric Top ranking articles. The most significant finding was that for Studies in Human Society, a statistically significant correlation between the use of Code Glosses and AAS was evident with a large effect size and small sample size. Finding a statistically significant result with a small sample size is very difficult as that is only likely to occur if the effect size is large whereas obtaining statistically significant results with a large sample often arises from the higher power linked with larger sample sizes, even with small effect sizes [90]. Code Glosses help readers understand the meaning and importance of the research by defining and elaborating on the information, demonstrating the writer’s understanding of shared subject knowledge with readers and the latter’s understanding of ideas, ultimately serving a persuasive function [76]. Readers can understand the significance of particular information in the way intended by the writer through expanding on issues [13]. In other words, elaborated ideas are associated with AAS, suggesting that this is a key persuasive feature required to gain high attention from readers, especially in news and Facebook.

Another significant finding was that for Information and Computing Sciences, a strong relationship between the use of Evidentials and AAS was observed. Readers from news and Twitter are more likely to refer to other texts to facilitate their interpretation of the text, thereby increasing the persuasiveness of the text. For example, readers were referred to previous work in the form of citations for more details. Citations are crucial to gaining credibility of new claims by providing support for arguments and showing that one is aware of the mainstream research [13]. While it is true that our findings depart from Hyland [91] who noted that Evidential Markers were used more often in research articles in the soft disciplines as opposed to those of the hard disciplines, it has to be emphasized that we investigated Altmetric Top ranking research article abstracts only rather than focusing solely on the use of metadiscoursal devices.

In addition, a positive correlation with a small effect size was noted between the use of Transitions, Frame Markers and Self-mentions and AAS in the Medical and Health Sciences. Our results also revealed a significant positive correlation between Transitions and the number of news mentions in AAS and a positive correlation between the use of Self-Mentions and the number of Twitter mentions in AAS. Transitions and Frame Markers represent internal logical connections in a text and are clear features of academic arguments that enable readers to not only comprehend the text but persuade them of the writer’s reasoning, thereby serving as tools of persuasion [13]. Self-mentions enable writers to assert their voice while fostering a credible scholarly identity, and enhancing the persuasiveness of research articles[13, 29]. Obtaining research funding for the Medical and Health Sciences has become increasingly competitive [92, 93], and therefore, writers often promote their contributions and the novelty and impact of their research by using Self-mentions, which in turn generates a high AAS. Our findings depart from Hyland’s [13] who found that Self-mentions were much more likely to be found in research articles in humanities and social sciences as opposed to those from science disciplines. Yet Hyland focused on the metadiscoursal devices in research articles whereas our study was concerned with the relationship between the use of Self-mentions and AAS.

In sum, our findings are in line with Zahedi and Haustein’s [69] study in that document characteristics (e.g. title length, number of pages) of research articles were associated with both altmetrics (Mendeley in particular) and citations. More importantly, we found that some metadiscourse markers, which increase the persuasiveness of texts, are intricately intertwined with AAS, and these devices are exhibited in different academic disciplines in distinct ways.

### Trends in the use of metadiscourse in papers garnering high public attention from 2014 to 2018

The Altmetric Top ranking article abstracts relied on the deployment of a wide range of metadiscourse markers to persuade and grab the attention of readers, especially on new media platforms. Our study reveals a significantly higher use of interactive than interactional metadiscourse in abstracts with high AAS. Previous studies investigating research articles from seven leading journals in Microbiology, Marketing, Astrophysics and Applied Linguistics [31] and postgraduate doctoral dissertations [39,90] also found that more interactive than interactional forms of metadiscourse were used but these studies focused on the complete research article/dissertation. The predominant use of interactive metadiscourse in our study highlights the importance of facilitating the reading process by showing the discourse organization and explaining propositional meanings and connections [13], and in particular, Transitions were frequently used to structure arguments.

Regarding interactive metadiscourse, Transitions and Evidentials were the most frequently used markers in abstracts with high AAS from 2014–2018. Transitions focus on the logical connections between ideas so that the text is coherent and convincing, while Evidentials refer to the previous literature, showing the novelty of the writer’s contribution and indicating a familiarity with the literature in the field, thereby contributing to the persuasiveness of arguments[13]. Our results are consistent with Hyland’s [94] and Hyland and Tse’s [39] analysis of metadiscourse used in masters and doctoral dissertations whereby they found that Transitions, Evidentials and Hedges were more commonly used than other devices.

However, our findings reveal a falling trend in the use of Evidentials from 2014 to 2018. This might be because most Medical and Health Sciences abstracts place emphasis on the need for the research, results found, value and impact of the research rather than highlighting previous research which may make ideas difficult to comprehend by the public and policy-makers, while all this salient information can make scientific knowledge more accessible to both the public and academic peers on social media. Our findings coincide with Author’s [4] study on research article abstracts, in which it was revealed that the Life Sciences discipline, which is similar to Medical and Health Sciences, adopted the move structure of introduction, results and conclusion with scant elaboration on the previous literature.

For interactional metadiscourse in Altmetric Top ranking article abstracts from 2014–2018, Self-mentions, Hedges and Boosters were frequently used. Our findings are congruent with Crismore and Farnsworth[27], Abdi [28] and Afros and Schryer [29] although these researchers only examined the use of metadiscourse in different genres (i.e. science and social sciences texts). The use of Self-mentions involves writers making a deliberate choice to assert their stance to their academic community and readers and making their voices heard. Prior research has shown that self-mentions were exhibited prominently in doctoral dissertations of research students whereby writers asserted their scholarly identity and obtained support for their ideas[29, 39, 94]. As our study placed emphasis on gaining attention from readers through linking AAS with metadiscourse markers in abstracts, we found support for the notion that Self-mentions can enhance the persuasiveness of abstracts when writers claim that they themselves have found novel findings or made original contributions so as to gain support from readers.

Hedges present claims with caution since writers can expect objections, so they express ideas with modesty while allowing readers to follow the writers’ claims, and this plays a crucial role in persuasion[13, 28]. On the other hand, Boosters convey certainty of claims, and put forward a single interpretation of findings, demonstrating confidence and persuasiveness[2, 28]. In fact, our findings are not completely aligned with Hyland[13], who found that the soft disciplines such as humanities and social sciences tended to use both more Boosters and Hedges than that in the hard sciences because soft disciplines are more interpretive. We could attribute this to the fact that most of our abstracts came from Medical and Health Sciences, whereby the importance of gaining the attention of readers through the use of especially Boosters and also Hedges has soared due to the competitive nature of obtaining grants and funding for research[92]. In addition, this discipline can sometimes be interpretive and inferential too, in which theories are suggested and then tested, and persuading academic peers and readers about the value and originality of the research is needed for academic advancement, which often will require the employment of Boosters and Hedges[4, 7].

Another noteworthy finding is the drop in the employment of Attitude Markers, which convey emotions like excitement, importance and agreement, using attitude verbs, adverbs and adjectives[13]. This is likely because Attitude Markers would often be viewed by most people as expressing the writer’s subjective attitude rather than focusing on the objectivity of claims/arguments, rendering the arguments or findings as not rigorous enough, and thereby less persuasive. Despite a decline in their use, these markers were still exhibited in Altmetric Top ranking article abstracts, indicating that they play an essential role in persuading readers. This may not be too surprising since readers, particularly the public, are possibly drawn to writers’ views or attitude on the research topic (i.e. peripheral cues of persuasion) instead of just the quality of arguments and objective data (i.e. central cues of persuasion)[9, 10]. Writers also use them to guide their readers’ understanding of what they perceive is important, surprising, and valuable or vice versa; for example, Darwin in Origin used Attitude Markers to achieve these communicative aims [95].

## Conclusion, implications, limitations and further research

In summary, our study is one of few to reveal that there is an association between metadiscourse and AAS in that a higher frequency of metadiscourse markers was evident in Altmetric Top ranking article abstracts from 2014–2018. Indeed, metadiscourse serves as a peripheral cue to persuade readers[10], helping writers to engage readers and facilitating the latter to interpret the text in ways that they will view it as convincing. Specifically, our study contributes to the literature in highlighting that there are variations in the use of metadiscourse in articles receiving high public attention from different disciplines in that most articles in the Medical and Health Sciences and social sciences fields tend to use more metadiscourse markers and have high AAS. Our study confirms Bondi’s [96] and Lindeberg’s [3]arguments favoring the use of specific disciplines in teaching academic literacy. Both interactive and interactional metadiscourse are necessary to render the research article abstract persuasive as revealed in the findings.

In terms of academic research, our work contributes to how rhetorical choices are made by researchers in article abstracts in each distinct discipline to gain readers’ attention on new and social media platforms. Concerning application, our results offer insights for researchers from different disciplines, who would benefit from being cognizant of the use of particular metadiscourse markers to enhance the persuasiveness of their abstracts given that it is much more difficult to get papers published in top-tier journals and obtain grants nowadays. It is suggested that researchers can construct their abstracts in such a way that they conform to the use of metadiscourse in specific disciplinary communities with the expectations of the target audience on social media. By doing so, researchers can make their abstracts more convincing to read and raise the interest generated, potentially leading to a higher successful rate of publication and funding.

Concerning tips on writing abstracts that are more likely to gain attention on new and social media platforms, it is suggested that Transitions, Evidentials, Self-mentions, Hedges, Boosters and Attitude Markers should be employed more frequently by science disciplines. However, for Medical and Health Sciences, a considerable use of Transitions, Frame Markers and Self-mentions is suggested with less usage of Evidentials and Attitude Markers. Furthermore, the increased use of Evidentials is highly recommended for Information and Computing Sciences and Code Glosses are recommended for Studies in Human Society or social sciences disciplines.

With regards to pedagogical implications, it is observed that English as a Foreign Language (EFL) and English for Academic Purposes (EAP) books often present metadiscourse in a piecemeal way and courses on EAP often do not give adequate attention to metadiscourse because they only include a limited number of markers [26, 76]. It is therefore important for students to be taught metadiscourse using tasks that would help them practice writing in their respective disciplines. Students need to have a sense of reader awareness and know how to engage readers, so tasks should be designed based on authentic texts. Metadiscourse has to be presented in a way that illustrates it plays a persuasive role in the interaction between the writer, readers and the text although it might just serve as a peripheral cue in persuasion.

Admittedly, limitations of this study include the fact that interdisciplinary and intradisciplinary variations could have possibly affected the results and therefore, discipline-specific studies with larger sample sizes should be conducted. However, even in medical sciences studies with large sample sizes, one has to treat with caution results with small effect sizes which might be statistically significant but lack clinical relevance [93]. Further research could focus on the use of different metadiscourse markers in different academic communities and genres and how they are related to AAS, as well as how the use of metadiscourse contributes to the goals of obtaining funding for research or acceptance of publications. Research on cultural variations in the use of metadiscourse can potentially illustrate differences and similarities between writers, which in turn can inform second language teaching.

## Supporting information

S1 Data (XLSX)
